# Novel synthetic benzimidazole-derived oligosaccharide, M3BIM, prevents ex vivo platelet aggregation and in vivo thromboembolism

**DOI:** 10.1186/s12929-016-0245-4

**Published:** 2016-02-17

**Authors:** Ting-Lin Yen, Ming-Ping Wu, Chi-Li Chung, Wen-Bin Yang, Thanasekaran Jayakumar, Pitchairaj Geraldine, Chih-Ming Chou, Chia-Yau Chang, Wan-Jung Lu, Joen-Rong Sheu

**Affiliations:** Graduate Institute of Medical Sciences and Department of Pharmacology, College of Medicine, Taipei Medical University, 250 Wu-Hsing St., Taipei, 110 Taiwan; Department of Obstetrics and Gynecology, Chi-Mei Medical Center, Tainan, Taiwan; Department of Medical Research and Translational Laboratory, Research Department, Taipei Medical University Hospital, 252 Wu-Hsing St., Taipei, 110 Taiwan; Genomics Research Center, Academia Sinica, Taipei, Taiwan; Department of Animal Science, School of Life Sciences, Bharathidasan University, Tiruchirappalli, Tamil Nadu India; Department of Biochemistry, College of Medicine, Taipei Medical University, Taipei, Taiwan; Hemophilia Center, Department of Pediatrics, Taipei Medical University Hospital, Taipei, Taiwan

**Keywords:** Arterial thrombosis, Benzimidazole-derived oligosaccharide, Cerebral infarction, Platelet aggregation

## Abstract

**Background:**

Thrombus formation, a phenomenon primarily related to increased platelet activation, plays a key role in cardiovascular and cerebrovascular diseases. Although the established antiplatelet agents, such as aspirin and clopidogrel, have been shown to be beneficial in treating thromboembolic diseases, they have considerable limitations. Hence, the development of more effective and safe antithrombotic agents is necessary to satisfy a substantial unmet clinical need. In recent years, the favorable properties of imidazole-related drugs have prompted medicinal chemists to synthesize numerous novel therapeutic agents. The chemical structure of the benzimidazole backbone has proven antiplatelet properties. Moreover, synthetic oligosaccharides have exhibited antiplatelet properties. Therefore, we developed a new aldo-benzimidazole-derived oligosaccharide compound, M3BIM, for achieving a stronger antiplatelet effect than the drugs which are being used in clinical aspects. We investigated the effects of M3BIM on platelet activation ex vivo and its antithrombotic activity in vivo.

**Results:**

M3BIM (10–50 μM) exhibited a more potent activity in inhibiting platelet aggregation stimulated by collagen than it did in inhibiting that stimulated by thrombin in washed human platelets. The M3BIM treatment revealed no cytotoxicity in zebrafish embryos, even at the highest concentration of 100 μM. In addition, M3BIM inhibited the phosphorylation of phospholipase Cγ2, protein kinase C (PKC), and mitogen-activated protein kinases (MAPKs; extracellular signal-regulated kinase 2 and c-Jun N-terminal kinase 1), and markedly reduced the ATP-release reaction and intracellular calcium mobilization in collagen-activated platelets. By contrast, M3BIM showed no effects on either collagen-induced p38 MAPK and Akt phosphorylation or phorbol 12, 13-dibutyrate-induced PKC activation and platelet aggregation. Moreover, the M3BIM treatment substantially prolonged the closure time in human whole blood, and increased the occlusion time in mesenteric microvessels and attenuated cerebral infarction in mice. For the study of anticoagulant activities, M3BIM showed no significant effects in the prolongation of activated partial thromboplastin time and prothrombin time in mice.

**Conclusion:**

The findings of our study suggest that M3BIM is a potential therapeutic agent for preventing or treating thromboembolic disorders.

## Background

Thrombus formation plays a key role in the pathogenesis of ischemic changes in various organs and systems of the body such as arterial thrombosis and cerebrovascular diseases. This phenomenon is primarily related to increased platelet activity and intravascular thrombus formation [1]. The involvement of platelets in hemostasis is associated with their ability to adhere and aggregate; release of the storage granule content; adsorption, deposition, and transportation of biologically active substances; and endothelial supporting functions [2]. When the vascular endothelium is disrupted, platelets adhere to the damaged intima and undergo activation. During activation, platelets release several mediators [including ADP and thromboxane A2 (TxA2)], which draw additional platelets toward the injured endothelium, causing the initial platelet monolayer to thicken. TxA2 is a crucial secondary mediator that is produced and released by stimulated platelets. The conversion of arachidonic acid (AA) into TxA2 is mediated by cyclooxygenase-1 and can be irreversibly inhibited by aspirin, which remains the standard drug for preventing cardiovascular diseases [3]. The use of antiplatelet drugs for various manifestations of activation of the platelet component of hemostasis can reduce the risk of thrombotic complications. Although the established antiplatelet agents, such as aspirin, clopidogrel, and tirofiban, have been shown to be beneficial in treating thromboembolic diseases, they have considerable limitations [4, 5]. Therefore, there is a great unmet clinical need for developing more effective and safe antithrombotic agents. Because the recent literature has emphasized the vital role of TxA2 in several pathologies, drugs that target the TxA2 pathway in platelets have emerged, including various TxA2 receptor antagonists (S-18886) [6], TxA2 synthase inhibitors (furegrelate) [7], and compounds combining both functions (ridogrel) [8]. However, the clinical efficacy of these inhibitors remains unclear.

**Fig. 1:**
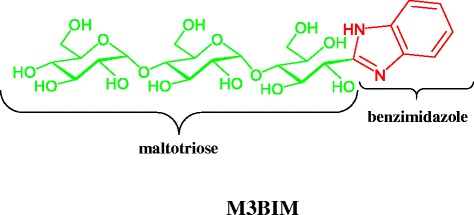
Chemical structure of benzimidazole-derived oligosaccharide (M3BIM)

Benzimidazole derivatives belong to a group of molecules that can be used to synthesize new substances with various biological properties [9]. In recent years, the favorable therapeutic properties of imidazole-related drugs have prompted medicinal chemists to synthesize numerous novel therapeutic agents. Imidazole-containing compounds have various medicinal properties such as anticancer [10], antimicrobial [11], antibacterial [12], and antifungal properties [13]. Compounds with a chemical structure containing a benzimidazole backbone have been shown to suppress platelet aggregation by inhibiting TxA2 synthase activity [14, 15]. Moreover, during the past decades, clarifications of the mechanisms of heparin and progress in oligosaccharide chemistry have facilitated the development of synthetic oligosaccharides [16]. Synthetic oligosaccharide polymers contain between three and ten simple sugars. Synthetic oligosaccharides also exhibit antiplatelet properties [17]. Encouraged by these observations to discover new biologically active benzimidazole-oligosaccharide compounds, we developed a new aldo-benzimidazole-derived oligosaccharide compound, M3BIM, from D-maltotriose (Fig. 1) to achieve stronger antiplatelet effects. This study provides the first evidence that the novel compound M3BIM can be developed further into a new class of antiplatelet agents.

## Methods

### Materials

Collagen (type I), luciferin-luciferase, arachidonic acid (AA), U46619, PDBu, heparin, prostaglandin E1 (PGE1), 2,3,5-triphenyl tetrazolium chloride (TTC), and thrombin were purchased from Sigma (St. Louis, MO, USA). Fura 2-AM, an intracellular calcium indicator, was purchased from Molecular Probes (Eugene, OR, USA). The anti-phospho-p38 MAPK Ser182 monoclonal antibody (mAb) was purchased from Santa Cruz Biotechnology (Santa Cruz, CA, USA). The anti-p38 MAPK, anti-phospho-JNK (Thr183/Tyr185), and anti-p44/42 ERK (1/2) mAbs and anti-PLCγ2, anti-phospho (Tyr759) PLCγ2, the anti-phospho-(Ser) PKC substrate, anti-JNK and anti-phospho-p44/p42 ERK (Thr202/Tyr204) polyclonal antibodies (pAbs) were purchased from Cell Signaling (Beverly, MA, USA). The anti-phospho-Akt (Ser473) and anti-Akt mAbs were purchased from Biovision (Mountain View, CA, USA). Anti-pleckstrin (p47) pAb was purchased from GeneTex (Irvine, CA, USA). The anti-α-tubulin mAb was purchased from NeoMarkers (Fremont, CA, USA). A hybond-P PVDF membrane, an enhanced chemiluminescence Western blotting detection reagent, the horseradish peroxidase (HRP)-conjugated donkey anti-rabbit immunoglobulin G (IgG), and the sheep anti-mouse IgG were purchased from Amersham (Buckinghamshire, UK). The Dade Behring PFA-100 CEPI and CADP test cartridges were obtained from Siemens Healthcare (Erlangen, Germany).

### Preparation and purification of M3BIM from maltotriose

According to the method described by Lin et al. [18], the compound M3BIM was prepared and purified through the oxidative condensation of maltotriose. Briefly, a mixture of D-maltotriose monohydrate (50.4 mg, 0.1 mM) and o-phenylenediamine (21.6 mg, 0.2 mM) was stirred with iodine (25 mg, 0.1 mM) in 3.0 mL of acetic acid for 30 h. The reaction was completed in 6 h as indicated by a subsequent thin layer chromatography analysis: (acetone/ethyl acetate/water/acetic acid, 60:30:20:1) Rf) 0.44; [R] 25 D + 55.40 (c 1.0, H2O). The reaction mixture was triturated with ethyl acetate to obtain precipitates, which were collected through filtration using a nylon membrane filter. The aldo-naphthimidazole products prepared using the aforementioned processes were essentially pure for characterization. The crude product was purified through C18 reversed-phase silica gel column chromatography (methanol/water, 1 to 30 % as gradient) to yield the product M3BIM (30 mg, 51 % yield, C24H36N2O15, yellowish foam). The freshly obtained M3BIM was dissolved in DMSO and stored at 4 °C.

### Platelet aggregation

This study was approved by the Institutional Review Board of Taipei Medical University and conformed to the directives of the Helsinki Declaration. All human volunteers who involved this study provided informed consent. Human platelet suspensions were prepared as previously described [19]. The human blood samples were obtained from adult volunteers, who refrained from the use of drugs or other substances that could interfere with the experiment for at least 14 days prior and mixed with an acid-citrate-dextrose solution. After centrifugation, the platelet-rich plasma was supplemented with 0.5 μM PGE1 and 6.4 IU/mL of heparin. A Tyrode’s solution containing 3.5 mg/mL of bovine serum albumin (BSA) was used to make final suspension of washed human platelets. The final concentration of Ca2+ in Tyrode’s solution was 1 mM. The platelet aggregation test was performed by using lumiaggregometer (Payton Associates, Scarborough, ON, Canada) as previously described [19]. Various concentrations of M3BIM or the solvent control (0.5 % DMSO) were preincubated into platelet suspensions (3.6 × 108 cells/mL) for 3 min before adding agonists (i.e., collagen). The extent of platelet aggregation was calculated as a percentage of the control (absence of M3BIM) of light-transmission units, after the reaction was preceded for 6 min. For tests of ATP release assay, 20 μL of a luciferin-luciferase mixture was added 1 min before the addition of the agonist, and the amount of ATP release was compared with that released by the control.

### Measurement of intracellular [Ca2+]i mobilization using Fura 2-AM fluorescence

The amount of intracellular calcium concentration [Ca2+]i was determined with Fura 2-AM as described previously [19]. Briefly, citrated whole blood was centrifuged at 120 × g for 10 min, and the collected supernatant was incubated with 5 μM Fura 2-AM for 1 h. Human platelets were prepared as described in the previous section. The Fura 2-loaded platelets were washed and pre-incubated with M3BIM in the presence of 1 mM CaCl2, and subsequently stimulated with collagen. The Fura 2 fluorescence was measured in a spectrofluorometer (Hitachi FL Spectrophotometer F-4500, Tokyo, Japan) with excitation wavelengths of 340 nm and 380 nm, and with the emission wavelength of 510 nm.

### Zebrafish toxicity test

Zebrafish (Danio rerio) were obtained from the zebrafish core facility of Taipei Medical University and maintained at 28 °C on a 14 h light/10 h dark cycle. All animal procedures were approved by the Taipei Medical University Institutional Animal Care and Utilization Committee. The zebrafish embryos were incubated at 28 °C and the developmental stages were determined using The Zebrafish Book [20]. Wild-type embryos were treated with various concentrations of M3BIM at 20 h postfertilization for 144 h to evaluate the toxic effects of M3BIM on the zebrafish embryos. Fifteen dechorionated embryos were treated with 2 mL of M3BIM (10, 20, 50, or 100 μM) or a solvent control (0.5 % DMSO) in a 24-well chamber. The treated embryos were observed at 2, 3, 4, 5, or 6 dpf. At 6 dpf, the percentage of embryos exhibiting developmental abnormalities and the survival rate were determined. During the exposure period, photographs of the embryos were observed under an Olympus IX70-FLA inverted fluorescence microscope (Olympus, Tokyo, Japan). The images were taken using a SPOT digital camera system (Diagnostic Instruments, Sterling Heights, MI, USA) and assembled using the Image J program [21].

### Immunoblotting

Washed platelets (1.2 × 109 cells/mL) were preincubated with various concentrations of M3BIM or a solvent control (0.5 % DMSO) for 3 min, followed by the addition of collagen to trigger platelet activation. The reaction was stopped, and the platelets were immediately resuspended in 200 μL of a lysis buffer. Samples containing 80 μg of protein were separated through 12 % SDS gel electrophoresis, and the proteins were electrotransferred to the PVDF membranes by using a Bio-Rad semidry transfer unit (Hercules, CA, USA). The blots were blocked with Tris-buffered saline in Tween 20 (TBST; 10 mM Tris-base, 100 mM NaCl, and 0.01 % Tween 20) containing 5 % BSA for 1 h and probed with various primary antibodies. The membranes were incubated with HRP-linked anti-mouse IgG or anti-rabbit IgG (diluted 1:3000 in TBST) for 1 h. An enhanced chemiluminescence system was used to detect immunoreactive bands, and their optical density was quantified by using Bio-profil Biolight software, Version V2000.01 (Vilber Lourmat, Marne-la-Vallée, France).

### Platelet function analysis in whole blood

The Dade Behring PFA-100 System (Marburg, Germany) was used to analyze platelet function [22]. Cartridges containing CADP- and CEPI-coated membranes were preincubated with various concentrations of M3BIM or the solvent control (0.5 % DMSO) for 2 min. Whole blood aliquots (0.8 mL/cartridge) were applied to the cartridges before the contents were exposed to high shear flow conditions (5000 to 6000/s). The CT was defined as the time required for a platelet plug to occlude the aperture in the collagen membrane [22].

### Measurement of sodium fluorescein-induced platelet thrombus formation in mice mesenteric microvessels

Male ICR mice (6 weeks) were anesthetized using a mixture containing 75 % air and 3 % isoflurane maintained in 25 % oxygen, and the external jugular vein was cannulated with a PE-10 tube for administering the dye and drugs intravenously [23]. Venules (30–40 μm) were irradiated at wavelengths < 520 nm to produce a microthrombus. Two doses of M3BIM (12 and 24 mg/kg) were administered 1 min following sodium fluorescein (15 μg/kg) administration and the time required for the thrombus to occlude the microvessel (occlusion time) was recorded. In this experiment, the method of thrombogenic animal model was conformed to the Guide for the Care and Use of Laboratory Animals (NIH publication no. 85–23, 1996).

### MCAO-induced focal cerebral ischemia in mice

Male C57/BL6 (6 weeks) mice were used in this study. All mice were clinically normal and free of apparent infections or inflammation, and showed no neurological deficits. The animals were anesthetized with a mixture of 95 % O2 and 5 % CO2 gasses containing 3 % isoflurane. The rectal temperature was maintained at 37 ± 0.5 °C. The right middle cerebral artery (MCA) was occluded as described by Hsiao et al. [23]. Briefly, the right common carotid artery was exposed and a 6–0 monofilament nylon thread coated with silicon was then inserted from the external carotid artery into the internal carotid artery until the tip occluded the origin of the MCA. After the closure of the operative sites, the animals were allowed to awaken from the anesthesia. During another brief period of anesthesia, the filament was gently removed after 30 min of MCAO. An observer blinded to the identity of the groups assessed neurological deficits at 1 and 24 h after reperfusion (before sacrifice) by using the forelimb akinesia (also called postural tail-hang) test, and the spontaneous rotational test was used for evaluating the ischemic insult [24]. Animals not showing behavioral deficits at the aforementioned time points after reperfusion were excluded from the study.

The mice were sacrificed through decapitation after 24 h of reperfusion. The brains were cut into 2-mm-thick coronal slices. Each stained (2 % TTC) brain slice was drawn using a computerized image analyzer (Image-Pro Plus). The calculated infarction areas were then compiled to obtain the infarct volumes (mm3) per brain. Infarct volumes were expressed as a percentage of the contralateral hemisphere volume by using the following formula to compensate for edema formation in the ipsilateral hemisphere: [area of the intact contralateral (left) hemisphere] − [area of the intact region of the ipsilateral (right) hemisphere] [23]. All animals were allocated into four groups: a sham-operated group, a solvent control (DMSO) group, and groups treated with 12 or 24 mg/kg (i.p.) of M3BIM.

### Ex vivo coagulation time assays

M3BIM (24 mg/kg), solvent control (0.5 % DMSO), or a phosphate-buffered saline (PBS) was injected (i.p.) in male ICR mice (6 weeks) for 30 or 60 min. The animals were then anesthetized using a mixture containing 75 % air and 3 % isoflurane maintained in 25 % oxygen. Whole blood (0.45 mL) was collected by cardiac puncture and transferred into plastic tubes containing 0.05 mL of 3.2 % citrate and was gently mixed. Specimens were centrifuged to obtain platelet-poor plasma. Activated partial thromboplastin time (APTT) and prothrombin time (PT) were obtained on a coagulation analyzer (Diagnostica Stago, Asnieres, France) using standard reagents according to the manufacturer’s instructions.

### Statistical analysis

The experimental results are expressed as the means ± SEM and are accompanied by the number of observations (n). Values of n refer to the number of experiments, and each experiment was conducted using different blood donors. Paired and unpaired Student’s t tests were used to determine significant differences in the occlusion time and MCAO-induced cerebral ischemia in mice, respectively. The differences between the groups in other experiments were assessed using ANOVA. When the ANOVA indicated significant differences among the group means, the groups were then compared using the Student-Newman-Keuls method. A value of P < 0.05 indicated statistical significance. Statistical analyses were performed using SAS Version 9.2 (SAS Inc., Cary, NC, USA).

## Results

### Inhibitory effects of M3BIM on platelet aggregation in washed human platelets

**Fig. 2:**
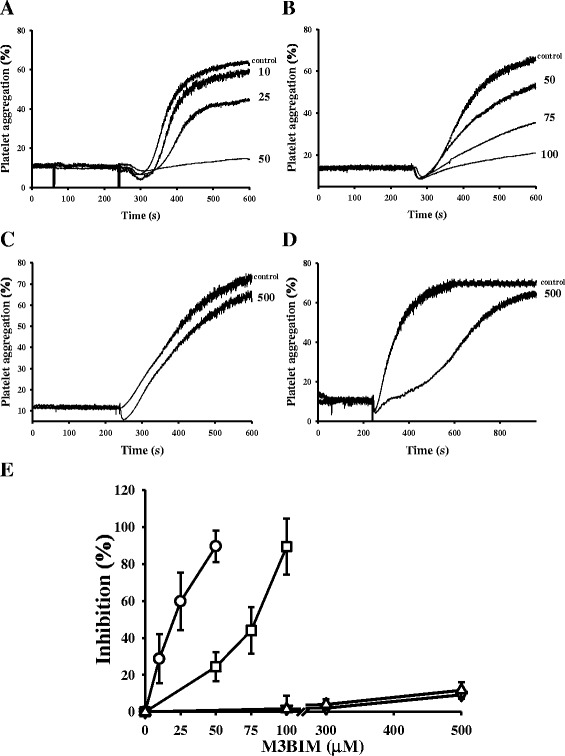
Inhibitory effects of M3BIM on agonist-induced platelet aggregation in washed human platelets. Washed human platelets (3.6 × 108 cells/mL) were preincubated with M3BIM (10–500 μM) or a solvent control (0.5 % DMSO) and subsequently treated with 1 μg/mL of collagen **a**, 0.05 IU/mL of thrombin **b**, 120 μM arachidonic acid **c**, or 0.5 μM U46619 **d** to stimulate platelet aggregation. The concentration-response curves of M3BIM in inhibiting platelet aggregation stimulated by collagen (○), thrombin (□), arachidonic acid (△) and U46619 (▽) are shown in **e**, and data are presented as the means ± SEM (n = 4)

As shown in Fig. 2a-b, M3BIM (10, 25, and 50 μM) concentration-dependently inhibited platelet aggregation in washed human platelets stimulated by 1 μg/mL of collagen. M3BIM exhibited similar inhibitory activity at a high concentration (100 μM) stimulated by 0.01 U/mL of thrombin (Fig. 2b). Moreover, M3BIM exhibited a slight inhibitory activity of platelet aggregation at very high concentration (500 μM) stimulated by 120 μM of AA (Fig. 2c) or 0.5 μΜ of U46619, a prostaglandin endoperoxide (Fig. 2d), indicating that M3BIM had no significant effects on thromboxane A2/prostaglandin E1 (TP) receptors or TP-synthesizing enzymes. The 50 % inhibitory concentration (IC50) values of M3BIM for platelet aggregation induced by collagen and thrombin were approximately 25 μM and 70 μM (Fig. 2e), respectively. M3BIM showed more potent activity in inhibiting collagen-stimulated platelet aggregation than it did in inhibiting thrombin-stimulated platelet aggregation. The solvent control (0.5 % DMSO) did not significantly affect platelet aggregation (Fig. 2a-b). In subsequent experiments, 1 μg/mL of collagen was used as an agonist for platelet activation.

### Influence of M3BIM toxicity in zebrafish embryos and ATP-release reaction and intracellular [Ca2+]i mobilization in washed human platelets

**Fig. 3:**
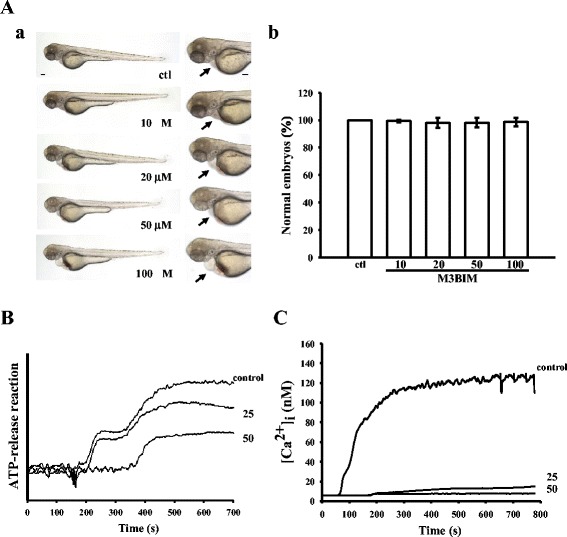
Influence of M3BIM on zebrafish embryonic development, and the ATP-release reaction and relative [Ca2+]i mobilization in human platelets. Wild-type zebrafish embryos (arrows) at 6 day postfertilization exposed to various concentrations of M3BIM (10, 20, 50, and 100 μM) or a solvent control (0.5 % DMSO) (scale bar 100 μm) (**a**,***a***). Statistical graphs (**a**,***b***) show the means ± SEM (n = 15). For other experiments, washed platelets (3.6 × 108 cells/mL) were preincubated with M3BIM (10, 20, 50, and 100 μM) or the solvent control (0.5 % DMSO), and 1 μg/mL of collagen was then added to stimulate either the ATP-release reaction (**b**) or to induce relative [Ca2+]i mobilization (**c**). Profiles (**b** and **c**) are representative of four independent experiments

To assess the M3BIM toxicity potential at the organism level, zebrafish, a versatile in vivo vertebrate model used in many areas of biological investigation, were used for this study. We evaluated the toxicity of M3BIM at ranges of 10–100 μM in wild-type zebrafish embryos treated for 6 days postfertilization (dpf). The results obtained from this assay showed no significant phenotypic differences between solvent control (0.5 % DMSO)- and M3BIM-treated zebrafish embryos throughout the experiment (n = 15) (Fig. 3aa-b). Notably, no developmental defects or decreases in viability (Fig. 3aa) were observed in the zebrafish embryos in the presence of M3BIM, event at the highest concentration of 100 μM. Furthermore, the aggregation curves of platelets that were preincubated with 100 μM M3BIM for 10 min and subsequently washed twice with Tyrode’s solution showed no significant differences from those of platelets preincubated with the solvent control (0.5 % DMSO) under equivalent conditions (data not shown). These results indicate that the inhibitory effects of M3BIM on platelet aggregation are reversible and noncytotoxic.

Platelet activation by several agonists is associated with the release of granular contents (e.g., release of ADP/ATP, calcium, and serotonin from dense granules) resulting in ample platelet aggregation. In the present study, we observed that M3BIM (25 and 50 μM) concentration-dependently inhibited both the ATP-release reaction (Fig. 3b) and relative [Ca2+]i mobilization (Fig. 3c) in washed human platelets stimulated by 1 μg/mL of collagen. As shown in Fig. 3c, collagen induced a relative increase in [Ca2+]i that was concentration-dependently inhibited by 25 and 50 μM M3BIM (resting, 19.0 ± 2.7 nM; collagen-activated, 132.7 ± 12.5 nM; 25 μM, 47.1 ± 22.8 nM; 50 μM, 25.5 ± 11.2 nM).

### M3BIM’s regulatory activity on phospholipase Cγ2 and protein kinase C activation

**Fig. 4:**
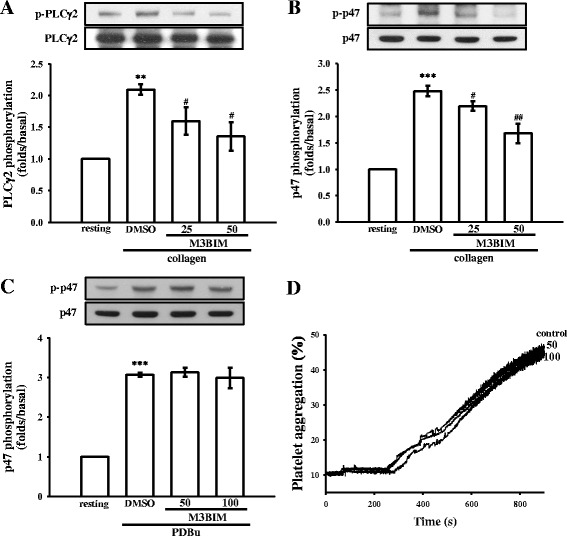
Regulatory effects of M3BIM on PLCγ2 and PKC activation in platelets. Washed platelets were preincubated with 25 or 50 μM M3BIM or a solvent control (0.5 % DMSO) and subsequently treated with 1 μg/mL of collagen or 150 nM PDBu to induce either PLCγ2 **a** or PKC activation (p47 phosphorylation) **b** and **c** and platelet aggregation **d**. Data are presented as the means ± SEM (n = 4). ** P < 0.01 and *** P < 0.001, compared with the resting platelets; # P < 0.05 and ## P < 0.01, compared with the platelets receiving the solvent control. Profiles **d** are representative of four independent experiments

The phospholipase C (PLC) enzyme hydrolyzes phosphatidylinositol 4,5-bisphosphate to generate the secondary messengers inositol 1,4,5-trisphosphate (IP3) and diacylglycerol (DAG). IP3 triggers intracellular Ca2 + i mobilization and DAG activates protein kinase C (PKC)-inducing protein phosphorylation (p47 protein; pleckstrin) and the ATP-release reaction in activated platelets [25]. We thus investigated the influence of M3BIM on phosphorylation of the PLCγ2-PKC signaling cascade. M3BIM treatment at concentrations of 25 and 50 μM evidently reduced PLCγ2 phosphorylation in collagen-activated platelets (Fig. 4a). Platelet stimulation by various agonists induces PKC activation and the subsequent phosphorylation of p47 proteins. A protein with an apparent molecular weight similar to that of p47 (47 kDa) was predominantly phosphorylated in collagen- and (150 nM) phorbol 12, 13-dibutyrate (PDBu, a PKC activator)-activated human platelets (Fig. 4b-c) compared with the proteins in non-activated platelets. M3BIM treatment at 25 and 50 μM reduced the evident collagen-induced p47 phosphorylation (Fig. 4b), but not PDBu-induced p47 phosphorylation, even concentration up to 100 or 200 μM (Fig. 4c). In addition, neither 50 nor 100 μM M3BIM significantly affected PDBu-induced platelet aggregation (Fig. 4d), indicating that M3BIM inhibits PKC activation through the upstream regulator PLCγ2.

### Inhibitory effect of M3BIM on Akt and mitogen-activated protein kinase activation

**Fig. 5:**
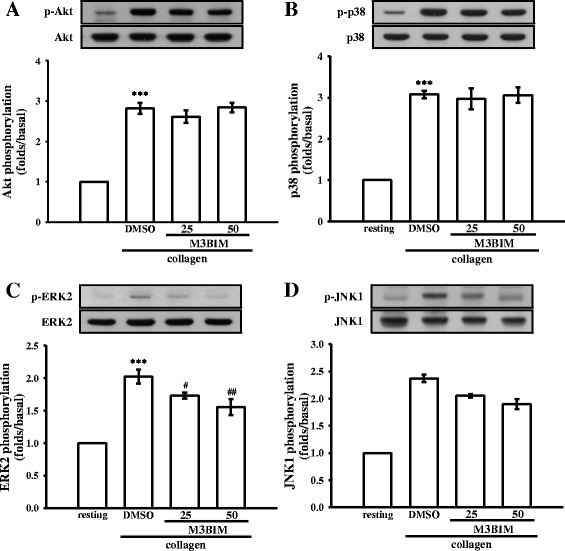
Effects of M3BIM on Akt, p38 MAPK, ERK2, and JNK1 phosphorylation in collagen-activated platelets. Washed platelets (1.2 × 109 cells/mL) were preincubated with 25 or 50 μM M3BIM or a solvent control (0.5 % DMSO) and subsequently treated with 1 μg/mL of collagen to induce platelet activation. The platelets were collected and the subcellular extracts were analyzed to determine the levels of Akt **a**, p38 MAPK **b**, ERK2 **c**, and JNK1 phosphorylation **d**. Data are presented as the means ± SEM (n = 4). *** P < 0.001, compared with the resting platelets; # P < 0.05 and ## P < 0.01, compared with the platelets receiving the solvent control

To investigate the inhibitory mechanisms of M3BIM in platelet activation, we detected several signaling molecules, such as Akt and mitogen-activated protein kinases (MAPKs). Akt is a Ser-Thr kinase with pleiotropic effects on cell survival, growth, and metabolism. MAPKs, including extracellular signal-regulated kinases 1 and 2 (ERK1/2), c-Jun N-terminal kinases 1 and 2 (JNK1/2), and p38 MAPK, control major cellular responses in eukaryotic organisms and contribute to cell proliferation, migration, differentiation, and apoptosis. M3BIM (25 and 50 μM) had no effects on inhibition of Akt or p38 MAPK phosphorylation (Fig. 5a-b); however, M3BIM (25 and 50 μM) concentration-dependently inhibited both ERK2 and JNK1 phosphorylation (Fig. 5c-d), indicating that the inhibition of the ERK2 and JNK1 signaling pathway is a crucial process in M3BIM-mediated inhibition of platelet activation.

### Ex vivo and in vivo studies of the role of M3BIM in thrombus formation and cerebral infarction

**Fig. 6:**
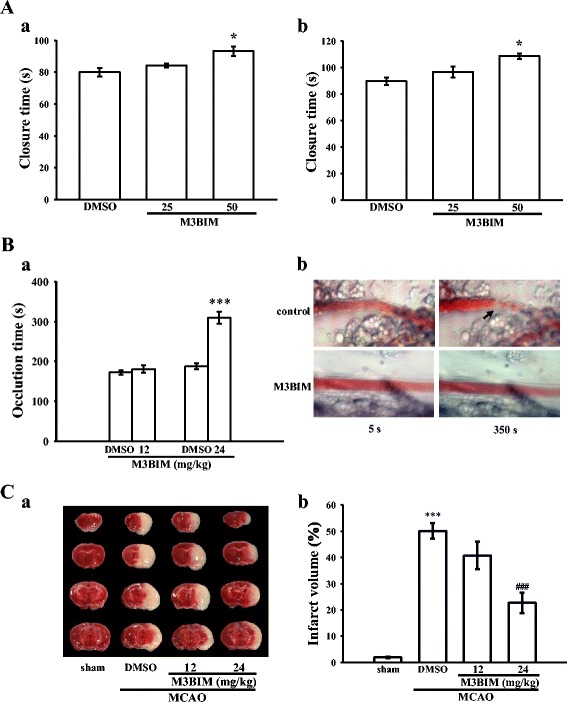
Protective effects of M3BIM on the closure time according to the PFA-100 analysis and thrombotic platelet plug formation in the mesenteric venules of mice, and middle cerebral artery occlusion (MCAO)-induced cerebral infarction in mice. (**a**) Shear-induced platelet plug formation in whole blood was determined by recording the (***a***) closure time of collagen/ADP (CADP) and (***b***) closure time of collagen/epinephrine (CEPI) as described in the Methods. (**b**) The mice were administered an intravenous bolus of a solvent control (0.5 % DMSO) or M3BIM (12 or 24 mg/kg), and the mesenteric venules were irradiated to induce microthrombus formation (occlusion time, a). Microscopic images (×400 magnification) of the solvent control- and M3BIM (24 mg/kg)-treated groups were recorded at 5 and 350 s after irradiation (***b***). The arrow indicates platelet plug formation. (**c**) Digital photographs (***a***) and dose–response bars (***b***) show the infarct region in brain sections stained with 2 % TTC 24 h after MCAO. Mice were injected with the solvent control or M3BIM (12 or 24 mg/kg, i.p.) before the onset of MCAO, and the infarct volume was compared with that of the sham group, as described in the Methods. The infarct volumes are expressed as a percentage of the contralateral hemisphere. Data (**a**-**c**) are presented as means ± SEM (A, n = 6; B-C, n = 8). * P < 0.05 and *** P < 0.001, compared with the DMSO group (**a** and **b**) or the sham group (**c**); ###P < 0.001, compared with the DMSO group (**c**)

In the present study, shear-induced platelet plug formation in whole blood was tested ex vivo. The PFA-100 instrument was used to mimic the in vivo conditions of blood vessel injury, in which platelets were exposed to a high shear rate to record the time required for platelet aggregation to occlude an aperture in a collagen-coated membrane. The closure times (CTs) of collagen/ADP (CADP) and collagen/epinephrine (CEPI) membranes in whole blood treated with a solvent control (0.5 % DMSO) were 80.0 ± 2.7 and 89.8 ± 2.9 s (n = 6), respectively (Fig. 6aa-b). Treatment with 50 μM M3BIM significantly increased the CTs of CADP (25 μM, 84.2 ± 1.3 s; 50 μM, 93.2 ± 2.9 s, n = 6) and CEPI (25 μM, 96.6 ± 4.2 s; 50 μM, 108.6 ± 2.0 s, n = 6) (Fig. 6aa-b), indicating that platelets were unable to adhere to collagen under flow conditions after the 50 μM M3BIM treatment. Furthermore, we investigated the effect of M3BIM on thrombus formation in vivo. The occlusion time in microvessels pretreated with 15 μg/kg of fluorescein sodium was approximately 180 s. When M3BIM was administered at 12 or 24 mg/kg after pretreatment with fluorescein sodium, the occlusion times were significantly prolonged at dose of 24 mg/kg compared with those of the solvent control groups (0.5 % DMSO, 172.5 ± 6.0 s vs 12 mg/kg, 180.6 ± 9.6 s, n = 8, P > 0.05; 0.5 % DMSO, 188.2 ± 7.2 s vs 24 mg/kg, 309.1 ± 15.3 s, n = 8, P < 0.001) (Fig. 6ba). After irradiation, a thrombotic platelet plug was observed in mesenteric microvessels at 350 s, but not at 5 s in the control group (Fig. 6bb). Upon the administration of 24 mg/kg of M3BIM, platelet plug formation was not observed at 5 or 350 s after irradiation (Fig. 6bb). The blood flow rate in the control venule was lower than that in the M3BIM-treated venule, because a platelet plug appeared at 350 s in the control venule (Fig. 6bb).

Platelet activation is relevant to various cerebrovascular disorders (e.g., ischemic stroke). Antiplatelet agents (e.g., acetylsalicylic acid and ticlopidine) have been shown to reduce the incidence of ischemic stroke in high-risk patients [26]. Therefore, we investigated the potential cerebroprotective effect of M3BIM after middle cerebral artery occlusion (MCAO) reperfusion in mice. All mice showed similar physiological values for rectal temperature, mean arterial blood pressure, plasma glucose, and hematocrit (%) before, during, and after MCAO (data not shown). Figure 6ca shows coronal sections of an ischemic brain (white area) from the sham control group and solvent control (0.5 % DMSO)-treated group, and coronal sections of the brain from mice pretreated with 12 and 24 mg/kg of M3BIM before ischemic insult. M3BIM treatment at the dose of 24 mg/kg (infarct volume, 22.7 ± 3.9 %, n = 8) but not at 12 mg/kg (infarct volume, 40.8 ± 5.3 %, n = 8), showed significant reductions in the infarct size compared with that in the mice treated with the solvent control (50.1 ± 3.0 %, n = 8) (Fig. 6cb).

### The influence of coagulation parameters in vitro by M3BIM

To further investigate whether the M3BIM also possesses anticoagulant activity, the APTT and PT was determined using mice blood, indicators for the efficacy of intrinsic and extrinsic pathways, respectively. As shown in the Table 1, M3BIM (24 mg/kg) had no significant prolongation of the APTT and PT values after 30 or 60 min treatment as compared with the PBS or solvent control (0.5 % DMSO), respectively. Positive control (warfarin, 3 mg/kg) markedly prolonged the APTT and PT values after 30 min treatment as compared with the PBS (APTT, warfarin, 80.6 ± 9.6 s vs PBS, 40.4 ± 2.7 s, n = 5, P < 0.01; PT, warfarin, 20.6 ± 2.9 s vs PBS, 13.1 ± 0.4 s, n = 5, P < 0.05; data not shown).

**Table 1:** The influence of M3BIM on activated partial thromboplastin time and prothrombin time in mice

	PBS	0.5 % DMSO	M3BIM	
	30 min	30 min	30 min	60 min
APTT (s)	40.4 ± 2.7	38.2 ± 4.3	39.9 ± 2.1	38.9 ± 1.5
PT (s)	13.1 ± 0.4	13.0 ± 0.4	11.8 ± 0.3	12.4 ± 0.1

M3BIM (24 mg/kg), solvent control (0.5 % DMSO), or a phosphate-buffered saline (PBS) was injected (i.p.) in mice for 30 or 60 min. The platelet-poor plasma was obtained for assay the activated partial thromboplastin time (APTT) and prothrombin time (PT). Data are presented as the means ± SEM (*n* = 8)

## Discussion

Our results show that M3BIM, a novel synthetic benzimidazole-derived maltotriose, exhibits potent antiplatelet activity ex vivo and effectively inhibits arterial thrombogenesis in vivo. Platelets are activated by a variety of physiological stimuli (e.g., thrombin and collagen). These agonists are thought to exert their effects by interacting with specific receptors on the platelet membranes. Thrombin is one of the most potent activators of platelets and its role in promoting thrombus formation has been clearly established. Thrombin activates platelets through multiple cell-surface receptors, including the glycoprotein (GP) Ib/V/IX complex and the protease-activated receptors (PARs) [27]. Of the 4 known PAR isoforms, PAR1, PAR3, and PAR4 constitute the active thrombin receptors on human platelets [28]. PAR1 and PAR4 are essential for thrombin-induced human platelet activation [29]. In addition, platelets adhere to the connective tissue protein collagen, with a resulting change in shape and the release of granules. The matrix protein collagen is present in the vascular subendothelium and vessel wall, and acts as a substrate for platelet adhesion; it is also an endogenous platelet activator. Among the platelet receptors known to interact directly with collagen, integrin α2β1 (GP Ia/IIa) and GP VI might contribute to the overall processes of platelet adhesion and activation [30–32]. In the present study, M3BIM inhibited platelet aggregation stimulated by collagen and thrombin, which indicates M3BIM was not effective on the specific/individual receptors of these agonists; however it may act via a common signal cascade against stimulated human platelets.

The activation of platelets by agonists, such as collagen, substantially alters phospholipase activation. The activation of PLC results in IP3 and DAG production, which activates PKC, inducing the phosphorylation of the p47 protein [25]. PKC activation enables particular responses that facilitate the transmission of specific activating signals in distinct cellular compartments. PLC enzymes can be classified into 6 families: PLCβ, PLCγ, PLCδ, PLCε, PLCζ, and PLCη [33]. The PLCγ family comprises isozymes 1 and 2. PLCγ2 is involved in collagen-dependent signaling in platelets [34]. In our study, M3BIM diminished collagen-induced PLCγ2-PKC activation; however, M3BIM exerted no direct effects on PKC activation, because it did not reduce PDBu-induced PKC activation or platelet aggregation, suggesting that M3BIM-mediated inhibition of platelet activation involves PLCγ2 downstream signaling. This result also explains how M3BIM comparatively efficacious in collagen-induced platelet aggregation than that of thrombin.

MAPKs are activated by specific MAPK kinases (MAPKKs or MEKs); specifically, MEK1/2 activates ERK1/2, MEK3/6 activates p38 MAPK, and MEK4/7 activates JNK1/2 [35]. Extensive studies have clarified that various growth factors and hormones that lead to cellular proliferation can activate the ERK1/2 signaling pathway via a Ras/Raf1/MEK1 signaling cascade [35]. In addition, various inflammatory cytokines and stress stimuli, which lead to cellular apoptosis or hypertrophy, activate JNK1/2 and p38 MAPK [35, 36]. ERK2, JNK1, and p38 MAPK were identified in platelets [37]. Little information is available on the molecular basis of the signaling pathway involved in ERK2 activation in platelets; however, a study reported that ERK2 activation depends on PKC and Src activation and increased myosine light chain kinase phosphorylation [38]. In addition, p38 MAPK plays a crucial role in platelet activation. Among the numerous downstream targets of p38 MAPK, cytosolic phospholipase A2, which catalyzes AA release to produce TxA2, is the most physiologically relevant [39]. JNK1 is the most recently identified MAPK in platelets, and its activation and role are therefore poorly defined. JNK1 activation was reported to trigger integrin αIIbβ3 activation [38]. Thus, MAPKs, particularly ERK2 and JNK1, but not p38 MAPK, appear to play a crucial role in M3BIM-mediated inhibition of platelet activation. Furthermore, Akt is a downstream effector of phosphoinositide 3-kinase (PI3-kinase) [40], and Akt-knockout mice have been shown to exhibit defects in agonist-induced platelet activation [41, 42]. In the present study, neither Akt nor p38 MAPK phosphorylation was inhibited by M3BIM. These findings are consistent with those of our previous study, where PI3-kinase/Akt and p38 MAPK were mutually activated in platelets [43].

After vascular endothelial cell injury, exposure to subendothelial collagen is the major trigger that initiates platelet adhesion and aggregation at the site of injury, followed by arterial thrombus formation. The PFA-100 instrument records the time required for platelet aggregation to occlude an aperture in a collagen-coated membrane. Platelet adhesion to collagen depends on flow conditions, and, in this study, platelets depleted of activity was unable to adhere to collagen under flow conditions. In a thrombosis study, the mesenteric venules were continuously irradiated by fluorescein sodium throughout the experimental period, leading to strong damage to the endothelial cells [19]. Cerebral ischemia restricted to the area of MCAO engenders focal metabolic disturbances that result in infarction, neuronal necrosis, and brain edema [23, 44]. In this study, M3BIM significantly reduced both arterial thrombosis and cerebral infarction; this effect may be mediated, at least partly, by inhibition of platelet activation. Furthermore, in the current study, we also examined the anticoagulant activity of M3BIM. There are two distinct pathways described for initiating blood coagulation, triggered by either vessel wall (extrinsic) or blood-borne (intrinsic) factors, which converge on a common pathway leading to thrombin generation and fibrin formation. Induction of fibrin clot formation through contact activation-mediated activation of factor XII in the intrinsic pathway is the basis of the APTT assay, a commonly used method for assessment of plasma coagulation in clinical settings [15]. The PT is an assay for the activation of coagulation through the extrinsic pathway triggered by factor VII coming into contact with tissue factor [15]. In the present study, we found that M3BIM had no effects on coagulant activities as it did not affect the APTT and PT values. In addition, the tail transection model of mice was used to examine the influence of M3BIM on bleeding time in vivo. Aspirin is the most effective antiplatelet drug prescribed for the prevention or treatment of cardiovascular and cerebrovascular diseases, whereas it has unwanted prolongation of the bleeding time. In the tail transection model of mice, aspirin was administered (i.p.) at 150 mg/kg after 30 min markedly prolonged the bleeding from 217.8 ± 19.0 s (PBS-treated control group; n = 8) to 428.1 ± 20.8 s (n = 8, P < 0.01; data not shown). The bleeding time of the M3BIM (24 mg/kg)-treated mice was slightly prolonged as compared with the solvent control (0.5 % DMSO)-treated mice (226.2 ± 19.5 s vs 288.6 ± 11.5 s, n = 8, P < 0.05; data not shown), indicating that the slightly prolonged the bleed time of M3BIM may be resulted, at least partly, from its antiplatelet activity.

## Conclusion

The findings of the current study reveal that M3BIM plays a novel role in inhibiting platelet activation. We report that M3BIM inhibits platelet activation by inhibiting the PLCγ2-PKC cascade and, subsequently, by suppressing ERK2 and JNK1 activation. These alterations reduce the level of [Ca2+]i and ATP-release reaction, and ultimately inhibit platelet aggregation. However, our experiments did not rule out the possibility that other as-yet-unidentified mechanisms might be involved in M3BIM-mediated platelet activation. Our findings suggest that a new class of synthetic benzimidazole-oligosaccharide derivative, M3BIM, is a novel potential therapeutic agent for preventing or treating thromboembolic disorders.

## Abbreviations

AA: arachidonic acid
APTT: activated partial thromboplastin time
BSA: bovine serum albumin
CADP: collagen/ADP
CEPI: collagen/epinephrine
CTs: closure times
DAG: diacylglycerol
dpf: day postfertilization
ERK: extracellular signal-regulated kinase
HRP: horseradish peroxidase
IP3: inositol 1,4,5-trisphosphate
JNK: c-Jun N-terminal kinase
MAPKs: mitogen-activated protein kinases
MCAO: middle cerebral artery occlusion
PARs: protease-activated receptors
PDBu: phorbol 12, 13-dibutyrate
PGE1: prostaglandin E1
PKC: protein kinase C
PLC: phospholipase C
PT: prothrombin time
TBST: Tris-buffered saline in Tween 20
TTC: 2,3,5-triphenyl tetrazolium chloride
TxA2: thromboxane A2
